# In Vitro/In Vivo Evaluation of a Portable Anesthesia Machine with an Oxygen Concentrator for Dogs Under General Anesthesia with Isoflurane

**DOI:** 10.3390/ani15070973

**Published:** 2025-03-27

**Authors:** Jungha Lee, Donghwi Shin, Taehoon Sung, Minha Kim, Changhoon Nam, Wongyun Son, Inhyung Lee

**Affiliations:** Department of Veterinary Clinical Sciences, College of Veterinary Medicine, Research Institute for Veterinary Science, Seoul National University, Seoul 08826, Republic of Korea; l2019jh@snu.ac.kr (J.L.); hwi4010@snu.ac.kr (D.S.); xogns7615@snu.ac.kr (T.S.); minha0221@snu.ac.kr (M.K.); skackdgns@snu.ac.kr (C.N.); carpeego@snu.ac.kr (W.S.)

**Keywords:** anesthesia machine, dog, isoflurane, oxygen concentrator, portable

## Abstract

This prospective, non-blinded study assessed the performance of a portable anesthesia machine with an oxygen concentrator (PAM_OC_) across various oxygen flow rates and vaporizer settings, incorporating both in vitro and in vivo experiments. The oxygen delivery test measured the time required to reach 90% fraction of inspired oxygen (FIO_2_) at various flow rates. The vaporizer test assessed the time to stabilize maximum fraction of inspired isoflurane (FIIso) concentration at various oxygen flow rate and vaporizer settings. In the in vivo test, six adult male Beagle dogs (11.4 ± 1.4 kg) were evaluated. The in vivo evaluation included monitoring physiological parameters during isoflurane anesthesia. The higher flow rates significantly reduced the time to plateau for FIO_2_ (*p* < 0.001). Maximum FIIso values were lower than the vaporizer dial settings, and increased oxygen flow rates significantly reduced the time required to reach target values (*p* < 0.001). Physiological parameters remained stable throughout anesthesia, confirming adequate oxygenation and anesthetic maintenance. The PAMoc, despite its lower pounds per square inch, yielded predictable outcomes consistent with those obtained in conventional anesthesia systems. These results demonstrated the viability of the PAMoc for anesthesia administration in the field and other challenging environments.

## Simple Summary

When using an oxygen concentrator as the oxygen source for a portable anesthesia machine, it is essential to assess whether the lower pressure affects oxygen delivery and anesthetic depth. This study investigated oxygen flow stability and whether higher flow rates reduce the time to reach target concentrations. Based on this, the study aimed to determine whether a portable anesthesia machine with an oxygen concentrator can maintain suitable conditions for anesthesia. The in vitro experiments assessed whether the oxygen concentration, despite not reaching the pounds-per-square-inch levels of an oxygen cylinder, could maintain stable levels of fraction of inspired oxygen and fraction of inspired isoflurane based on the flow rates. The in vivo experiments evaluated the safety and efficacy of a portable anesthesia machine with an oxygen concentrator for anesthesia administration in clinical use, using adult male Beagle dogs (11.4 ± 1.4 kg). The results confirmed that the portable anesthesia machine with an oxygen concentrator provided stable flow rates in vitro and maintained ventilation and physiological stability in vivo during anesthesia. The portable anesthesia machine with an oxygen concentrator is a potential reliable and accessible alternative to traditional anesthesia methods in difficult situations.

## 1. Introduction

In difficult situations, such as working in resource-poor environments, it can be challenging to maintain advanced medical equipment due to the limited availability of skilled personnel and resources, which can negatively impact healthcare quality [1,2]. However, the adoption of appropriate medical technology can provide solutions for those seeking to improve the quality of care administered [1,2]. The concept of appropriate medical technology has also been applied to anesthesia machines, leading to the development of simplified and cost-effective devices that can be used in resource-limited settings. One of the advancements in this area is the development of portable anesthesia machines (PAMs), designed to function effectively in difficult situations where conventional anesthesia machines may not be feasible [3,4,5,6,7]. These devices can be categorized based on whether they require an external oxygen source or operate as draw-over systems. Machines that do require an oxygen source can be further subcategorized based on whether they use oxygen cylinders or concentrators [8,9]. Draw-over systems are highly portable, although they are not recommended for patients weighing less than 10 kg owing to performance limitations. In addition, draw-over vaporizers are less accurate than plenum vaporizers [8,9]. Oxygen cylinders, which are commonly used, can be risky to transport and have a limited usage time [10]. Oxygen-cylinder-based PAMs are widely available, while devices utilizing oxygen concentrators are relatively uncommon. Among those that do exist, many are limited by their inability to provide oxygen flow rates exceeding 5 L/min or by their significant weight, which restricts their mobility to flat surfaces only [4,7].

**Figure 1:**
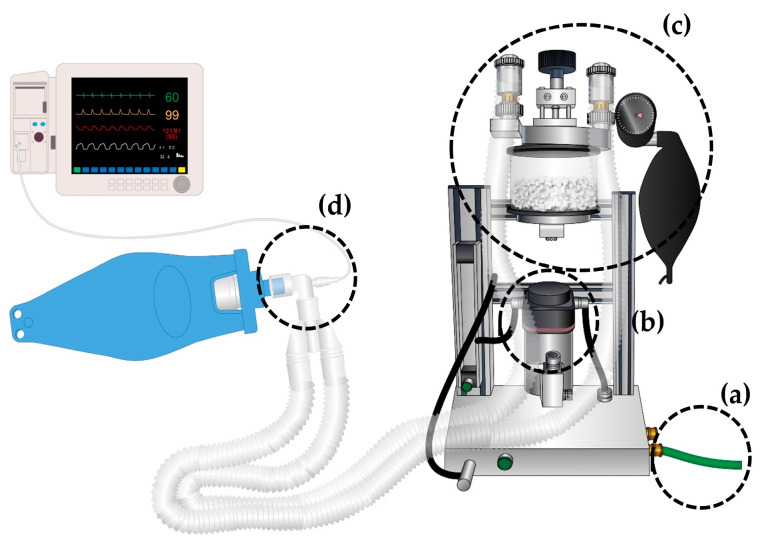
Schematic diagram of the portable anesthesia machine. (**a**) Oxygen supply line connected to an oxygen concentrator. (**b**) Plenum-type vaporizer for isoflurane administration. (**c**) Breathing circuit utilizing a rebreathing system and a passive scavenging system. (**d**) A gas sample line connected to the Y connector of the breathing tube, with measurements obtained using a sidestream capnography system.

To overcome these limitations, the authors have developed a portable anesthesia machine with an oxygen concentrator (PAM_OC_) as an alternative to traditional devices, particularly for use in difficult situations. To enhance the ease of use, the PAM_OC_ includes a rebreathing system with standard connectors, a conventional flowmeter, and a passive scavenging system [11] (Figure 1). The rebreathing system includes a reservoir bag (available in different sizes), unidirectional valves to regulate gas flow, and a CO_2_ absorber to remove exhaled carbon dioxide, ensuring efficient gas recycling and minimizing anesthetic gas waste. To improve portability and reduce overall weight, a ventilator was excluded from the design.

The oxygen concentrator is capable of delivering a fraction of inspired oxygen (FIO_2_) of 93% ± 3%, with oxygen flow rates of up to 10 L/min, and oxygen can be continuously supplied at a pressure of 6–10 pounds per square inch (PSI) as long as a power source is available. Anesthesia machines require an oxygen source capable of supplying a pressure of at least 50 PSI to function effectively [11]; however, oxygen concentrators are incapable of maintaining this pressure, raising concerns about their performance as an oxygen source for anesthesia machines.

This study evaluated the performance of the PAM_OC_, an anesthesia delivery system utilizing an oxygen concentrator instead of a high-pressure oxygen source, to determine whether it could maintain a stable oxygen flow rate and achieve target concentrations of oxygen (FIO_2_) and inspired isoflurane (FIIso) under varying flow conditions. In vitro experiments were conducted to assess the effect of different oxygen flow rates on the time required to reach target concentrations of FIO_2_ and FIIso. In vivo experiments were performed in six Beagle dogs to monitor physiological parameters during anesthesia and to assess whether the PAM_OC_ could maintain a stable anesthetic state during clinical use.

## 2. Materials and Methods

### 2.1. In Vitro Test

#### 2.1.1. Oxygen Delivery Test

The oxygen concentrator (JAY-10, Longfian Scitech Co., Ltd., Baoding, China) was connected to the PAM_OC_, and the time required to reach an FIO_2_ of 90% and sustain it for at least 1 min was assessed at various oxygen flow rates of 1, 2, 5, and 10 L/min (T1, T2, T5, and T10, respectively). An oxygen concentrator was warmed up before use. The measurements were repeated four times, and a statistical analysis was conducted to evaluate intergroup differences. For each measurement, a rebreathing system was set up by attaching a gas sample line (Sample Line Set 2.5 m, Dragerwork AG & Co., Lübeck, Germany) to the Y connector of the breathing tube (DAR™ Adult Breathing Circuit 2 L; Covidien™, Mirandola, Italy), thereby enabling continuous FIO_2_ sampling within it. The tube length was consistently maintained at 1.5 m. In this experiment, a test lung (Test Lung RP20, Foxxmed Ltd., Yilan, Taiwan) was used, although the procedure was conducted without performing ventilation, and the pop-off valve was kept fully open throughout the experiment. Sidestream capnography was used to continuously monitor the gas concentrations throughout the procedure (Figure 1). All flow rates and gas concentrations were evaluated using a patient monitor commonly used in hospitals (CARESCAPE Monitor B650, GE Healthcare, Helsinki, Finland). The patient monitor was regularly maintained and used in accordance with standard hospital procedures; however, additional calibration was not performed before the experiment. More specifically, at an oxygen flow rate of 5 L/min, FIO_2_ values were recorded at 10 s intervals to ensure stable and precise oxygen delivery.

Randomization and blinding were not implemented in this study, because the experimental design required precise control and systematic evaluation of oxygen flow rates under standardized conditions. To reduce potential bias, data collection was conducted automatically using monitoring equipment, thereby ensuring objective and accurate measurements of the FIO_2_ levels and the times to reach plateau.

#### 2.1.2. Vaporizer Test

A newly purchased plenum vaporizer (SHEI Temperature Compensated Vaporizer, Royal Medical Co., Ltd., Seoul, Republic of Korea) was used under continuous flow at various volumetric rates per minute. The vaporizer was calibrated by the manufacturer before sale. Isoflurane (Isotroy 100, Troikaa Pharmaceuticals Ltd., Ahmedabad, India) was selected as the inhalation anesthetic for this study due to its widespread clinical use. Before each test, a thorough leakage check of the anesthesia machine was performed to ensure that no gas could escape. The pop-off valve of the circuit remained open throughout the experiment. Similar to the oxygen delivery test, ventilation using a breathing bag was not performed. An oxygen concentrator was warmed up before use. This study compared two oxygen sources, an oxygen concentrator and an oxygen cylinder, with the cylinder serving as the control. Both were tested under the same flow conditions and vaporizer settings to assess their impact on FIIso.

The experimental method involved measuring the time to reach the maximum FIIso value and its corresponding concentration at different oxygen flow rates of 1, 2, 5, and 10 L/min, and the vaporizer settings were adjusted from 0% to 4%, reflecting clinically relevant concentrations. The maximum FIIso was defined as the point at which the FIIso approached the setting on the vaporizer dial and remained stable for at least 1 min. The measurement locations and methods were conducted in the same method as in the oxygen delivery test. These measurements were repeated four times for each flow rate (T1, T2, T5, and T10) and vaporizer setting (T1, T2, T3, and T4). After each measurement, the breathing tubes were flushed using positive pressure ventilation, and it was confirmed that the FIIso value on the monitor was zero before starting a new measurement. A statistical analysis was performed to evaluate differences between conditions.

Randomization and blinding were not implemented in this study, because the experimental design required precise control and systematic evaluation of oxygen flow rates under standardized conditions. To reduce potential bias, data collection was conducted automatically using monitoring equipment, thereby ensuring objective and accurate measurements of the FIIso levels and times to reach plateau.

### 2.2. In Vivo Test

This study was approved by the Institutional Animal Care and Use Committee of Seoul National University (SNU-230427-8). Six healthy adult male Beagles with an average weight of 11.4 ± 1.4 kg were enrolled in the study. Before anesthesia, each dog underwent a comprehensive preanesthetic evaluation, which included a physical examination, blood sampling for a complete blood count and serum chemistry analysis, and thoracic radiography. The dogs fasted for 6 h before the procedure and were allowed unrestricted access to water.

For sedation, medetomidine was administered intravenously at a dose of 2.5 μg/kg (Domitor, Pfizer Ltd., Bristol, UK), and anesthesia was induced using intravenous alfaxalone at a dose of 2 mg/kg (Alfaxan, Jurox Pty Ltd., Rutherford, Australia). Isoflurane was used for anesthesia maintenance, and the dogs were intubated using a cuffed endotracheal tube connected to an appropriate breathing circuit. An intravenous infusion of Ringer’s lactate solution (Hartmann’s solution, 5 mL/kg/hr, Hartmann Injection 100 mL, Dai Han Pharm. Co., Ltd., Seoul, Republic of Korea) was administered throughout anesthesia. Spontaneous respiration was preserved, with the oxygen flow set at 2 L/min throughout the procedure, and the pop-off valve remained fully open. The vaporizer dial setting was increased from 0% to 3% and maintained for 10 min; it was subsequently reduced to 2% for the following 20 min, adjusted to 1% for 10 min, and finally set to 0% for an additional 10 min. The total duration of anesthesia did not exceed 60 min for any of the dogs. A 24-gauge intravenous catheter was inserted in the left or right dorsal pedal artery to allow for continuous monitoring of the mean arterial blood pressure (MAP). Physiological parameters, including the heart rate (HR) (beats per minute), respiratory rate (RR), peripheral capillary oxygen saturation (SpO_2_), MAP, end-tidal carbon dioxide partial pressure (PE’CO_2_), end-tidal fraction of isoflurane (FE’Iso) (percentage), and FIO_2_, were recorded every 10 min. Arterial blood samples were collected once within 10 min post-intubation and before the end of anesthesia. Blood samples were analyzed using a blood gas analyzer (ABL90, Radiometer, København, Denmark).

After the procedure, the dogs were closely monitored until they were able to walk unassisted, at which point they were returned to their cages for recovery. Randomization and blinding were not conducted, because the study was designed to evaluate physiological responses under consistent anesthetic protocols and each dog served as its own control. Investigator involvement was necessary to conduct real-time adjustments and collect data, making blinding impractical. To minimize bias, the objective parameters were recorded automatically using a monitor to ensure consistency and reliability across all measurements.

### 2.3. Statistical Analyses

Statistical analyses were conducted using IBM SPSS Statistics, Version 29 (IBM Corp., Armonk, NY, USA), with the significance level set at *p* < 0.05. Normality of the data distributions was evaluated using the Shapiro–Wilk test. The results indicated that both the oxygen delivery test and vaporizer test data did not follow a normal distribution (*p* < 0.05). Consequently, statistical analyses were performed using non-parametric methods to ensure the validity of the findings. Prior to this study, preliminary experiments were conducted, including oxygen delivery and vaporizer tests. Based on the data from these experiments, the effect size was determined to be 0.8, which indicated a large effect size [12]. Applying this value, the required sample size was determined using G*Power software (Version 3.1, Heinrich Heine University, Germany); assuming an alpha error of 5% and a power of 80%, the required sample size was four for each group.

For the oxygen delivery test, the Friedman test was used to analyze the relationship between the oxygen flow rate and the time required to reach an FIO_2_ of 90%. Post hoc comparisons were conducted using the Wilcoxon signed-rank test to identify significant intergroup differences, with the significance level adjusted to 0.83% according to the Bonferroni method.

For the vaporizer test, the Friedman test was employed to analyze the time required to reach the maximum FIIso at different oxygen flow rates while maintaining fixed vaporizer concentrations. Post hoc pairwise comparisons were performed using the Wilcoxon signed-rank test to determine whether significant differences existed between groups, with the significance level adjusted to 0.83% according to the Bonferroni method. Results are reported as the median (range) and mean ± standard deviation.

## 3. Results

### 3.1. In Vitro Test

#### 3.1.1. Oxygen Delivery Test

**Figure 2:**
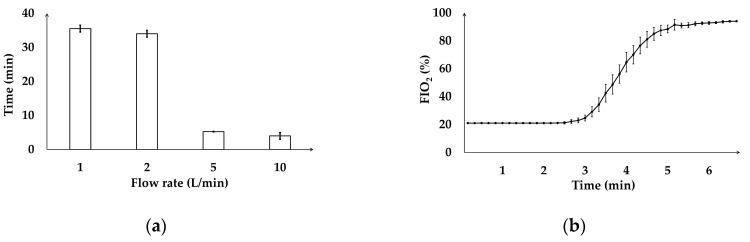
Oxygen delivery test. (**a**) The time (min) required to reach 90% FIO_2_ depending on the O_2_ flow rate (L/min). (**b**) Change over time (min) of FIO_2_ (%) produced by the oxygen concentrator at a flow rate of 5 L/min. Error bars indicate range. FIO_2_, fraction of inspired oxygen.

Oxygen delivery testing was conducted in an environment with a temperature range of 23.1–27.5 °C. The oxygen concentration required to reach a plateau was determined to be 90% for each oxygen flow rate, which was consistent with the manufacturer’s specification of 93 ± 3%. At lower flow rates of 1 and 2 L/min, the times (T1, T2) to achieve the maximum oxygen concentration were 36.05 (35.72–36.37) min and 34.55 (34.27–34.97) min, respectively. In contrast, higher flow rates of 5 and 10 L/min shortened the times (T5, T10) needed to reach 90% FIO_2_, requiring just 5.25 (5.20–5.30) min and 4.40 (4.32–5.00) min, respectively (Figure 2a). At an oxygen flow rate of 5 L/min, the FIO_2_ remained at 21%, which corresponded to the air in the testing room, during the first 2.64 ± 0.21 min; after this period, a rapid increase in the FIO_2_ was observed, reaching 90% at approximately 5.18 ± 0.28 min. The FIO_2_ increase rate slowed as the level approached 93%, indicating that a stable plateau was achieved (Figure 2b).

The statistical analyses revealed significant differences among the groups in terms of the time required to reach 90% FIO_2_ (*p* = 0.008). Post hoc comparisons using the Wilcoxon signed-rank test revealed a significant difference between T1 and T10 (*p* = 0.002), whereas all other compared values (T1 and T2, T1 and T5, T2 and T5, T2 and T10, T5 and T10) were not statistically different (*p* = 0.414, *p* = 0.04, *p* = 0.218, *p* = 0.02, and *p* = 0.273, respectively).

#### 3.1.2. Vaporizer Test

**Figure 3:**
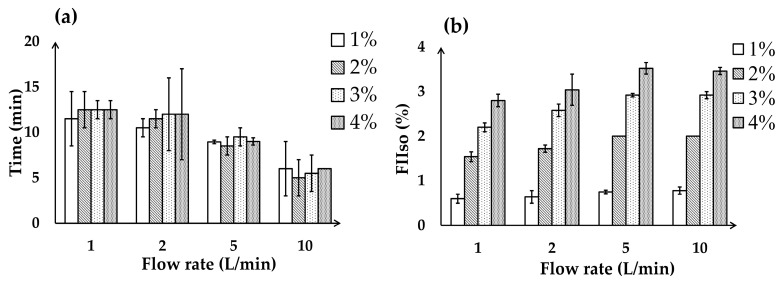

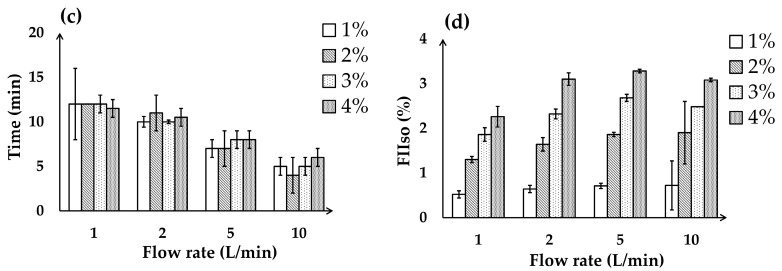
Vaporizer test. (**a**) The time (min) required to reach the maximum FIIso (%) based on the O_2_ flow rate (L/min) using oxygen concentrator. (**b**) Maximum FIIso (%) based on the O_2_ flow rate (L/min) using oxygen concentrator. (**c**) The time (min) required to reach the maximum FIIso (%) based on the O_2_ flow rate (L/min) using oxygen cylinder. (**d**) Maximum FIIso (%) based on the O_2_ flow rate (L/min) using oxygen cylinder. Error bars indicate range. FIIso, fraction of inspired isoflurane.

The vaporizer tests were conducted in an environment with a temperature range of 22.5–24.4 °C. The time required to reach the maximum FIIso for each setting was measured (Figure 3a). During continuous gas flow, the FIIso was lower than the setting indicated on the vaporizer dial at all flow rates (1–10 L/min). For all vaporizer settings, increasing the oxygen flow rate resulted in a decrease of the time required to reach the maximum FIIso. A difference exceeding 0.5% between the setting indicated on the vaporizer and the actual measured FIIso was observed in 14 of 64 instances (21.8%). This difference was most pronounced at an oxygen flow rate of 1 L/min, which occurred in eight of fourteen instances (57.1%), as well as for vaporizer settings of 0 to 4%, which accounted for nine of fourteen instances (64.3%) (Figure 3b). In the experiment involving the use of an oxygen cylinder, the time required to reach the maximum FIIso concentration was similar to that observed using the oxygen concentrator (Figure 3c,d).

The analysis of the time required to reach the maximum FIIso at a fixed vaporizer dial setting with varying oxygen flow rates revealed a significant difference across all conditions, as determined by the Friedman test (*p* < 0.05) (Table 1). The post hoc analysis confirmed a significant difference between T1 and T10 at all vaporizer dial settings (1, 2, 3, and 4%) (*p* = 0.003, *p* < 0.001, *p* = 0.004, and *p* = 0.003, respectively). Additionally, at a vaporizer setting of 2%, significant differences were observed between T1 and T5 (*p* = 0.003), as well as T2 and T10 (*p* = 0.001).

### 3.2. In Vivo Test

**Figure 4:**
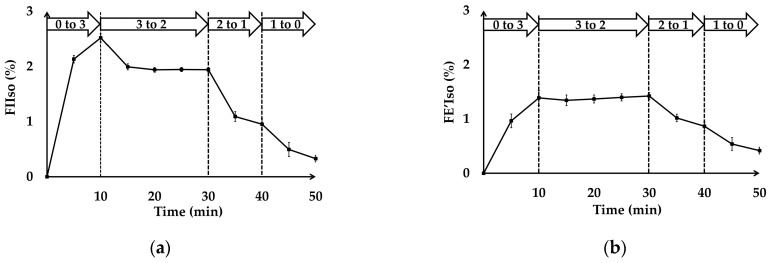
In vivo test. (**a**) The FIIso (%) for the six Beagle dogs over time (min). (**b**) The FE’Iso (%) for the six Beagle dogs over time (min). The vaporizer dial setting was increased from 0 to 3% and maintained for 10 min, reduced to 2% for the following 20 min, then adjusted to 1% for 10 min, and finally set to 0% for an additional 10 min. Error bars indicate standard deviations. FE’Iso, end-expiratory concentration of isoflurane; FIIso, fraction of inspired isoflurane. Open arrow: vaporizer set (%).

The oxygen flow rate was maintained at 2 L/min while the vaporizer settings were changed. At a constant oxygen flow rate of 2 L/min, the vaporizer settings were adjusted from 0% to 3% for the six dogs, and the FIIso and end-expiratory concentration of isoflurane (FE’Iso) concentrations were monitored over a 10 min period. When the vaporizer was set at 0 to 3%, the FIIso and FE’Iso reached mean levels of 2.51 ± 0.07% and 1.38 ± 0.14%, respectively, after 10 min. Subsequent reductions in the vaporizer settings resulted in corresponding decreases in the FIIso and FE’Iso. More specifically, when the vaporizer settings were adjusted from 3 to 2%, 2 to 1%, and 1 to 0%, the FIIso and FE’Iso were determined to be 1.94 ± 0.04% and 1.42 ± 0.06%, 0.95 ± 0.09% and 0.87 ± 0.08%, and 0.27 ± 0.14% and 0.34 ± 0.16%, respectively. Regardless of the vaporizer settings, the FE’Iso values consistently lagged behind the FIIso values and failed to reach the expected levels within a 10 min interval (Figure 4a,b).

Throughout the test, all physiological parameters remained stable. The Beagles’ HR, RR, PE’CO_2_, SpO_2_ (>95%), MAP (>60 mmHg), and FIO_2_ (91.75 ± 1.16%) were within the anticipated ranges and exhibited minimal fluctuations in response to changes in the vaporizer settings. The blood gas analysis confirmed that oxygenation was effectively maintained, with arterial partial pressure of oxygen values of 448.6 ± 54.3 mmHg within 10 min post-intubation and 479.3 ± 26.7 mmHg before the end of anesthesia. No abnormalities were detected in the arterial blood gas values. One of the six dogs exhibited premature arousal within 10 min after the vaporizer setting was adjusted from 1% to 0%, whereas the other five dogs recovered uneventfully following vaporizer shutdown.

## 4. Discussion

In the oxygen delivery test, FIO_2_ reached 90% and was sustained for 1 min before the experiment was concluded. At an oxygen flow rate of 5 L/min, FIO_2_ 90% was achieved in about 5 min. In the vaporizer test, FIO_2_ concentration was not measured; instead, the focus was on assessing the time required to reach the maximum FIIso value at different oxygen flow rates and vaporizer settings. The results showed that the time to reach the maximum FIIso varied depending on the O_2_ flow rate. The in vivo experiments further confirmed that the PAM_OC_ was able to maintain appropriate ventilation and physiological stability during anesthesia. Similar to the conventional method utilizing an oxygen cylinder, the PAM_OC_ achieved the target FIO_2_ and FIIso concentrations, and it did so more rapidly as the oxygen flow rate increased. Additionally, the PAM_OC_ demonstrated consistent performance in delivering and maintaining stable FIO_2_ and FIIso levels, suggesting its potential as a reliable alternative to conventional anesthesia methods in clinical settings.

The sensor was positioned at the end of the Y connector of the breathing circuit rather than directly at the outlet of the oxygen concentrator and vaporizer to assess the actual time required for the gas to reach an appropriate concentration at the patient end. In the oxygen delivery and vaporizer tests, the oxygen flow rate influenced the time required to reach the target FIO_2_ and FIIso levels. In the oxygen delivery test, at lower flow rates of 1 and 2 L/min, the time to reach the target concentration was substantially prolonged, whereas higher flow rates of 5 and 10 L/min markedly reduced this time. The Friedman test identified a significant difference only between T1 and T10, despite descriptive statistics indicating similarities between T1–T2 and T5–T10. This outcome is attributed to the rank-based nature of the test, where significance depends on relative ranking rather than absolute values. In the vaporizer test, the measured FIIso values were consistently lower than the vaporizer dial settings, likely due to anesthetic absorption within the anesthesia circuit, as reported in previous studies [11]. At low fresh gas flows (<1 L/min), the rebreathing system requires a longer equilibration period, which contributes to greater discrepancies between the vaporizer dial setting and the measured FIIso [13,14]. In the present study, the notable discrepancy observed at 1 L/min and a 4% vaporizer setting is likely not only due to reduced vaporizer output under low-flow conditions, but also because the gas concentration was measured at the Y connector via a gas sample line rather than at the common gas outlet.

The time to reach maximum FIIso was significantly influenced by oxygen flow rate rather than vaporizer settings, with higher flow rates achieving faster equilibration. Using high fresh gas flow rates at the beginning of anesthesia has been shown to facilitate quicker oxygen and anesthetic uptake, while also effectively eliminating nitrogen [13].

This study confirmed that the same principle applied to the PAM_OC_, despite the lower operational PSI of oxygen concentrators compared to conventional anesthesia machines. These results align with the expected outcomes observed with traditional anesthesia machines, further demonstrating that the PAM_OC_ performance characteristics do not differ from those of conventional systems. When using the PAM_OC_, it is recommended to pre-set oxygen flow rates to 5 L/min or higher and maintain this flow for at least 5 min before use on a patient to ensure adequate oxygen concentration.

The in vivo testing highlighted the connection between the vaporizer settings and both FIIso and FE’Iso concentrations. Although the FIIso adjusted rapidly in response to changes in the vaporizer settings, the FE’Iso consistently exhibited delayed responses, particularly at higher vaporizer settings. This delay might have been influenced by physiological factors such as alveolar ventilation or the uptake of isoflurane by tissues [15]. Throughout the experiment, the six dogs exhibited stable physiological parameters including HR, RR, MAP, and SPO_2_, indicating that the changes in vaporizer settings were generally well tolerated.

The PAM_OC_ possesses distinct features compared to those of existing PAMs and represents a novel alternative for portable anesthesia management. The design of the plenum vaporizer used in the PAM_OC_ incorporates features such as temperature and flow compensation, ensuring that the anesthetic concentration remains consistent despite fluctuations in environmental conditions or gas flow rates [9]. Moreover, the plenum vaporizer can be advantageous when administering anesthesia to animals with low body weights [8], because its precise calibration and agent-specific functionality enable stable anesthetic delivery without the variability often associated with draw-over systems. Furthermore, the plenum vaporizer’s ability to maintain its performance in low-flow systems optimizes gas utilization, making it a practical and efficient choice for portable setups [9,16].

Reliance on oxygen cylinders can be challenging owing to their weight, limited portability, and classification as hazardous materials, necessitating strict handling and storage protocols to reduce explosion risks. Oxygen cylinders also require specialized facilities approved by the Gas Safety Corporation for storage, which further complicates their use [10]. In addition, the amount of oxygen present in the cylinder determines the usable time. Therefore, it is important to accurately verify the remaining duration of use. For example, a standard 5 L oxygen cylinder can provide oxygen at a flow rate of 2 L/min for approximately 8 h [17]. As the oxygen flow rate increases, the available usage time decreases more rapidly; thus, monitoring the remaining oxygen level consistently is important. In contrast, oxygen concentrators offer a safer and more practical alternative by continuously supplying oxygen for as long as there is a power source available from which to draw energy. They also do not require refilling or specialized storage conditions, thereby significantly reducing the logistical burden [18]. Over extended periods, oxygen concentrators have also proven to be more cost-effective, because they eliminate the recurring expenses associated with cylinder refills and purchases [19]. High inspired oxygen levels, such as an 100% FIO_2_, are associated with adverse effects, including pulmonary atelectasis [20,21]. In contrast, lower oxygen levels can preserve lung aeration and enhance gas exchange [22]. Therefore, maintaining FIO_2_ levels < 100% during anesthesia may be beneficial for optimizing pulmonary function and minimizing the risk of oxygen-related complications. These features render oxygen concentrators not only more economical than cylinders but also more convenient and safer for long-term use in various clinical and portable settings.

To enhance portability, the PAM_OC_ was designed without a ventilator. An O_2_ flush valve is included, but due to insufficient pressure, it functions only for short durations rather than continuous use. Nevertheless, effective ventilation management has been achieved by optimizing spontaneous breathing in patients and by applying intermittent positive-pressure ventilation. Utilizing an oxygen concentrator capable of delivering flow rates up to 10 L/min can facilitate manual ventilation without challenges. Although incorporating a ventilator could enhance control, it would add considerable weight to the system and diminish its portability, which is an important consideration for the PAM_OC_. In veterinary anesthesia equipment, oxygen flush valves often lack the safety standards of human equipment, such as ISO 80601-2-13, allowing for excessively high flow rates that can cause dangerous pressure build-up within the breathing circuit [23,24]. Therefore, the inability to use the oxygen flush valve is not a serious drawback, because it eliminates the risk of pressure-related complications while maintaining safer ventilation management practices. PAM_S_ based on the draw-over system, as well as those using oxygen cylinders as the gas source, do not require electricity and have been used in settings without a power supply [6,7]. In contrast, the PAM_OC_ employs an oxygen concentrator requiring a stable electrical supply. This makes it suitable for use in environments where power is consistently available, such as mobile veterinary units, volunteer programs supported by generators, and field conditions like zoological institutions. Given its reliance on electricity, it is recommended that a bag–valve–mask resuscitator be prepared as a backup in case of unexpected power failure, to ensure continuous ventilation.

This study has a few limitations. First, performance of the PAM_OC_ over extended periods was not evaluated, because the focus was on short-term use under controlled experimental conditions. However, such conditions may not fully capture the variability and challenges encountered in routine clinical or field scenarios, potentially limiting the generalizability of the results. Second, the time required to reach the target concentration of both oxygen and isoflurane may vary depending on several factors, including breathing circuit size, system dead space, and manual ventilation application [25]. Additionally, when replicating the experiment, the time may differ based on the portable anesthesia machine. Third, as this study included only dogs, the applicability of the findings to other species is limited. Additionally, while FIIso and FE’Iso values were measured to assess inhalant anesthetic concentrations, these parameters alone are not sufficient to fully evaluate anesthetic depth. To address these limitations, future studies should explore the long-term performance and reliability of the PAM_OC_ in diverse clinical and field settings. Investigations involving different animal species are also essential for evaluating the broader applicability and effectiveness of this device.

## 5. Conclusions

The study findings confirmed that the PAM_OC_ can serve as a dependable alternative to traditional anesthesia machines by ensuring the stable delivery of flow rates required to achieve the FIO_2_ and FIIso levels essential for anesthesia. The in vitro experiments demonstrated that the PAM_OC_ has the capability to consistently achieve target oxygen and inhalation anesthetic concentrations, which can be attained more rapidly at higher oxygen flow rates. Furthermore, the in vivo experiments validated the efficacy of the device for maintaining appropriate ventilation and physiological stability during anesthesia.

## Figures and Tables

**Table 1:** Analysis of the time required to reach the maximum FIIso (%) at a fixed vaporizer dial setting (%) with varying O_2_ flow rates (L/min).

Vaporizer Dial (%)	Friedman Test	Wilcoxon Signed-Rank Test	*p*
1	*p* = 0.011	T1 and T2	0.584
		T1 and T5	0.055
		T1 and T10	0.003
		T2 and T5	0.171
		T2 and T10	0.014
		T5 and T10	0.273
2	*p* < 0.001	T1 and T2	0.175
		T1 and T5	0.003
		T1 and T10	<0.001
		T2 and T5	0.1
		T2 and T10	0.001
		T5 and T10	0.121
3	*p* = 0.011	T1 and T2	0.784
		T1 and T5	0.075
		T1 and T10	0.004
		T2 and T5	0.132
		T2 and T10	0.009
		T5 and T10	0.121
4	*p* = 0.011	T1 and T2	0.584
		T1 and T5	0.055
		T1 and T10	0.003
		T2 and T5	0.171
		T2 and T10	0.014
		T5 and T10	0.273

The vaporizer dial setting was tested at four levels (1, 2, 3, and 4%). FIIso, fraction of inspired isoflurane.

## Data Availability

The raw data supporting the conclusion of this article will be made available by the authors upon request.
